# Improved method for quantification of regional cardiac function in mice using phase-contrast MRI

**DOI:** 10.1002/mrm.23022

**Published:** 2011-06-14

**Authors:** Erica Dall'Armellina, Bernd A Jung, Craig A Lygate, Stefan Neubauer, Michael Markl, Jürgen E Schneider

**Affiliations:** 1 Department of Cardiovascular Medicine, University of OxfordOxford, United Kingdom; 2 Department of Radiology, Medical Physics, University Hospital FreiburgGermany

**Keywords:** mouse hearts, transmural wall motion, phase-contrast MRI, motion encoding, cine-MRI, ischemia–reperfusion

## Abstract

Phase-contrast magnetic resonance imaging is a technique that allows for characterization of regional cardiac function and for measuring transmural myocardial velocities in human hearts with high temporal and spatial resolution. The application of this technique (also known as tissue phase mapping) to murine hearts has been very limited so far. The aim of our study was to implement and to optimize tissue phase mapping for a comprehensive assessment of murine transmural wall motion. Baseline values for regional motion patterns in mouse hearts, based on the clinically used American Heart Association's 17-segment model, were established, and a detailed motion analysis of mouse heart for the entire cardiac cycle (including epicardial and endocardial motion patterns) is provided. Black-blood contrast was found to be essential to obtain reproducible velocity encoding. Tissue phase mapping of the mouse heart permits the detailed assessment of regional myocardial velocities. While a proof-of-principle application in a murine ischemia–reperfusion model was performed, future studies are warranted to assess its potential for the investigation of systolic and diastolic functions in genetically and surgically manipulated mouse models of human heart disease. Magn Reson Med, 2012. © 2011 Wiley Periodicals, Inc.

Genetically and surgically modified mice have become the animal models of choice for human cardiac disease. Fast, high-resolution, multiframe magnetic resonance imaging (cine-MRI) has been established in recent years as a routine tool in many research laboratories around the world to noninvasively assess global cardiac function (1–6). Left ventricular volumes and mass are measured from the cine images in end-diastolic and end-systolic phases of the heart, allowing for the calculation of cardiologically relevant parameters such as stroke volume, ejection fraction, or cardiac output. Three different techniques have been reported to characterize cardiac function in mice regionally and to measure transmural wall motion, and they are as follows: (A) myocardial tagging—a grid of signal voids is generated by modulating the magnetization with gradient fields (spatial modulation of magnetization) (7). The tags are then tracked throughout the cardiac cycle by a multiframe sequence. (B) Displacement encoding with stimulated echoes (DENSE (8)) is based on a stimulated echo sequence, in which the displacement of the myocardium is directly encoded in phase images. Both tagging and DENSE allow for the calculation of myocardial strain and have been applied to various mouse models of cardiac disease (9–14). Importantly, DENSE was initially limited to the application at a single time point within the cardiac cycle (typically end-systole (13, 14)) but has recently been extended to multiphase application in the mouse (15). (C) The third method, dubbed phase-contrast MRI or tissue phase mapping (TPM), encodes the velocity rather than displacement in the phase information of the MR signal. To the best of our knowledge, only one group has reported on the implementation of TPM for the assessment of myocardial function in mice (16–18). The first study by Streif et al. (16) used a 7-T MR system and encoded in-plane motion in a mid-ventricular slice only. Mean magnitude velocities, obtained in four regions (i.e., anterior, posterior, septal, and lateral walls), were quantified in normal and chronically infarcted hearts at four time points of the cardiac cycle. Work by Herold et al. (17) advanced this technique and obtained three-dimensional motion patterns in an apical, a mid-ventricular, and a basal slice in normal mice with very high-spatial resolution (98 μm in-plane and 0.6-mm slice thickness) on a 17.6-T MR system requiring rather long scan times of 30–40 min. Axial and angular velocities were again quantified at four time points of the cardiac cycle, and the local twist angle in apical and basal slices was calculated. Nahrendorf et al. (18) demonstrated the drastically reduced contraction and relaxation velocities in creatine kinase-deficient mice, using the technique implemented in Ref.^16^. All these studies used bright-blood contrast. However, it is well recognized that TPM in humans (at lower magnetic field strength) requires suppression of the dominant blood signal to provide an accurate measurement of myocardial velocities (19, 20). Blood suppression has also been shown to improve image appearance in tagging of mouse hearts (3).

This article aims to overcome the limitations described above. Specifically, we sought: (A) to investigate the feasibility of a comprehensive assessment of myocardial wall motion in three dimensions analogously to clinical practice at three levels in the heart (i.e., basal, mid-ventricular, and apical slices, respectively), using a conventional, commonly available preclinical MR system interfaced to a 9.4-T magnet; (B) to optimize the method with respect to temporal/spatial resolution and scan time; (C) to demonstrate the benefits of black-blood over bright-blood contrast in murine phase-contrast MRI; (D) to establish baseline values for regional motion patterns in mouse hearts, based on the American Heart Association 17-segment model (21); (E) to provide a detailed motion analysis of murine hearts for the entire cardiac cycle; (F) to characterize differences in epicardial and endocardial motion patterns; and (G) to use it in a murine ischemia–reperfusion model as a proof-of-principle application.

## MATERIALS AND METHODS

### MR Hardware

All MR experiments were carried out on a 9.4-T (400 MHz) MR system [Agilent Technologies, Santa Clara, CA (formerly Varian)] comprising a horizontal magnet (bore size = 210 mm), a VNMRS Direct Drive™ console, an actively shielded gradient system (1000 mT/m, rise time = 130 μs, od/id = 115/60 mm), and a quadrature-driven birdcage resonator (id = 33 mm; Rapid Biomedical, Rimpar, Germany).

### Animal Preparation

C57Bl/6 mice (5 female and 17 male, *n* = 22, 23.1 ± 1.8 g) were obtained from a commercial breeder (Harlan, UK) at least 1 week before the first imaging time point to allow naturalization to new surroundings. The mice were kept under controlled conditions for temperature, humidity, and light, with chow and water available ad libitum. Anesthesia was induced in an anesthetic chamber using 4% isoflurane in 100% oxygen. Animals were then positioned prone on dedicated mouse cradles and maintained at 1.5–2% isoflurane at 2 L/min oxygen flow throughout the MRI experiments. A 0.5-mL Eppendorff tube filled with 1% agarose (spiked with 2 mM Gd-DTPA) was placed next to the chest of the animal to correct background phase errors (22). Temperature was maintained at ∼37 °C using a warm air blanket placed below the animal. Cardiac and respiratory signals were continuously monitored using an in-house developed ECG and respiratory gating device (23).

In five C57Bl/6 mice (male, 27.9 ± 3.0 g), the left coronary artery was occluded for 45 min followed by reperfusion as described in Ref.^24^. The animals were subjected to phase-contrast MRI 24-h postsurgery.

All investigations conformed to Home Office Guidance on the Operation of the Animals (Scientific Procedures) Act, 1986 (HMSO) and to institutional guidelines.

### Phase-Contrast Imaging

The phase-contrast imaging sequence was based on a two-dimensional multiframe gradient echo sequence. Motion-compensating and -encoding gradients were combined with the imaging gradients as reported previously (16, 17). After positioning the mice in the magnet with the heart in the center and scouting for long- and short-axis orientations of the heart using a double-gated, segmented gradient-echo sequence, shimming and pulse calibrations were performed automatically before each experiment. Datasets were acquired on a basal, mid-ventricular and apical level with the following imaging parameters: FOV = 25.6 mm^2^, matrix size = 128 × 128, TE/TR = 2.1/4.6 ms, slice thickness = 1 mm, 15° sinc excitation pulse, number of averages NT = 2, venc_in-plane_ = 6 cm/s, and venc_through-plane_ = 8 cm/s. Velocity-encoded/compensated scans were acquired in an interleaved fashion. For each line in *k*-space, the motion-compensated acquisition was executed for all time frames of one cardiac cycle, followed by the three motion-encoded scans in three successive heartbeats before incrementing the phase-encoding gradient (i.e., next *k*-space line). A saturation module (total duration: 7.5 ms) at the end of each cine-train, which consisted of two 4-mm saturation slices, applied 4.5 mm above and below the imaging slice, followed by a crusher gradient, provided black-blood contrast. The remaining ventricle was covered with conventional bright-blood cine-imaging by turning off the motion-encoding and black-blood modules (NT = 1, TE = 1.1 ms). All sequences were ECG-triggered and respiratory-gated with steady-state maintenance during respiration [i.e., the sequence continued to run during respiration with the same timing (i.e., synchronized to the cardiac cycle), but no data were acquired, and all internal counters were not incremented—see Ref.^23^ for details]. The number of frames per cardiac cycle was determined by the heart rate, and the number of phase-encoding steps per respiration cycle—acquired in a segmented fashion (5)—was adapted to the respective respiratory rate. Each TPM-data set required 3–4 min of scan-time depending on heart and respiratory rates.

#### Sequence Validation

Velocity assessment by means of phase-contrast MRI was validated on a flow phantom with pulsatile flow using an MR-compatible flow-sensor (placed inside the magnet and outside the RF coil) before in vivo application. TPM experiments were conducted at three different flow rates (35, 40, and 45 mL/min) with venc's of 40, 50, and 60 cm/s, respectively. A trigger signal was derived from the flow curve and used to synchronize the MR experiment.

#### Bright versus Black-Blood Contrast and Reproducibility of Black-Blood Contrast

The benefit of black-blood over bright-blood contrast was investigated in a subset of mice (*n* = 6, 23.6 ± 1.2 g), where a mid-ventricular slice was additionally acquired with identical sequence parameters, but the saturation module at the end of the cine-train turned off. Typically, two more frames were obtained instead in this case. To exclude the inherent variability of the black-blood contrast technique, its reproducibility was assessed in three more mice (31 ± 4 g) by repeating the acquisition of a mid-ventricular slice with black-blood contrast.

#### Data Analysis

Reconstruction and analysis of data were performed offline using purpose-written *idl*-software (ITT, Boulder). All raw data were isotropically zerofilled by a factor of two and filtered (modified third-order Butterworth filter (25)) before Fourier transformation resulting in a reconstructed in-plane voxel size of 100 × 100 μm^2^.

##### TPM data

Three phase maps containing the motion-related velocity information in *x*-, *y*-, and *z*-directions were obtained by subtracting the respective motion-encoded scan from the phase reference (i.e., motion-compensated scan). Magnitude images (for segmentation) were calculated by adding the magnitude of all four scans. Phase maps and magnitude data were exported into binary format and read by a purpose-written software tool to analyze the TPM data in MATLAB (The Mathworks Inc., Natick). After manually delineating the epicardial and endocardial borders on a frame-by-frame and slice-by slice basis, the measured in-plane velocities (*v*_x_ and *v*_y_) were transferred into an internal polar coordinate system, positioned at the centre of mass of the left ventricle, and expressed as radial (*v*_r_) and circumferential (tangential, *v*_ϕ_) velocities. Furthermore, all velocities were corrected for bulk motion by subtracting global translational velocities from the local velocity components (19). Global cardiac motion patterns were analyzed by averaging the velocity components over the entire segmentation mask, resulting in velocity time courses for basal, mid-ventricular, and apical slices of each mouse. To facilitate a more detailed analysis of regional cardiac function based on the 17-segment model according to American Heart Association and American College of Cardiology recommendation (21), the left ventricle was partitioned at each time frame into six equiangular segments on basal/midventricular and four equiangular segments on an apical slice level. The 17th segment, which only contains the apex, was excluded from analysis. Based on the respective median radius, each segment was divided further into an epicardial compartment and an endocardial compartment. For a cumulative assessment of global and regional myocardial patterns, time courses (normalized to end-systole) were averaged over all mice for each slice and each velocity component as described previously (26).

The regional peak systolic and diastolic velocities measured in the ischemia–reperfusion hearts were normalized to the control hearts according to:


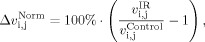
1

with *i* ∈ [radial longitudinal], and *j* ∈ [peak - diastole, peak - systole], respectively.

Twist angles and LV torsion were derived as described in Ref.^27^. The twist angle ϕ was calculated from the angular velocities *v*_rot_ for each segment according to:


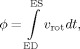
2

where the integration is carried from end-diastole to end-systole, and *v*_rot_ is given by:


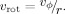
3

*r* is the median radius of the segment. Ventricular torsion was calculated for four segments (i.e., anterior, inferior, lateral, and septal walls, respectively) as:


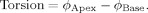
4

The sign convention for the various velocities used throughout the article follows Ref.^26^, and a positive radial velocity indicates contraction, i.e., the myocardium moves toward the center-of-mass. A positive in-plane rotation is defined as clockwise when viewed from foot-to-head direction. The longitudinal velocity is considered positive if the myocardium moves from base to apex.

##### Black- versus bright-blood and black- versus black-blood comparison

Two types of analyses were performed to assess agreement between bright- and black-blood contrast as well as reproducibility of the TPM method with black-blood contrast. The three global velocity components from corresponding time frames within the cine-train for each subject, obtained in a mid-ventricular slice, were subjected to (1) regression and (2) Bland–Altman analysis using Microsoft Excel 2008 for Apple MacIntosh. The total number of data points available for both analyses is equal to the sum over all time frames and animals. The null hypothesis that the slope in the regression analysis was equal to one was tested using a Student's *t*-test. A *P*-value of 0.05 was considered statistically significant. Furthermore, correlation coefficients were calculated by correlating mean radial velocities in 24 angular segments with the global velocity time course (28).

##### Global cardiac function

For each data set of the normal hearts, cardiac structural (i.e., left-ventricular mass, end-diastolic volume, and end-systolic volume) and functional parameters were obtained from TIFF images as described before (5), using Amira 4.1 (Visage Imaging GmbH, Berlin, Germany).

## RESULTS

### Validation

The flow rates in the validation experiment were quantified to 35.3, 40.6, and 44.7 mL/min and were therefore in excellent agreement with the preset flow rates. The accuracy of the flow rate at the pump was ±0.5 mL/min, whereas the standard deviation, derived from the phase noise in the experiments, for the flow measured with phase-contrast MRI was 0.01 mL/min.

### Black- versus Bright-Blood Contrast

**Fig. 1:**
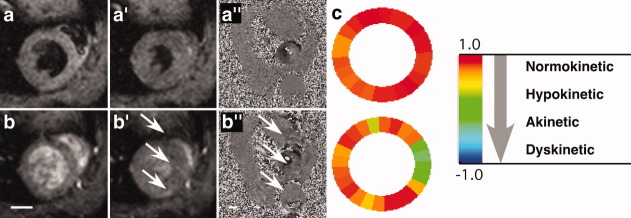
**a**–**a**″: Black-blood versus (**b**–**b**″) bright-blood magnitude and phase images, indicating low-level, blood flow–related artifacts in the septum (arrows in b′,b″). The unprimed panels correspond to end-diastole and the primed panels to end-systole. **c**: These artifacts may be interpreted as abnormal regional wall motion as illustrated by the correlation coefficient analysis. Scale bar: 2 mm. [Color figure can be viewed in the online issue, which is available at wileyonlinelibrary.com.]

Figure 1 exemplarily compares end-diastolic and end-systolic frames obtained from the same animal with black-blood (top row) and bright-blood (bottom row). Blood flow–related artifacts can clearly be observed along the phase-encoding direction in the bright-blood images and the corresponding phase maps (see arrows in Fig. 1b′,b″). Figure 1c shows the corresponding plots of the correlation coefficients indicating a greater variability in the bright-blood data caused by artifacts. Correlation coefficients close to 1 characterize a synchronous contraction/expansion over the entire LV as expected for a normal mouse heart and seen for the black-blood data in Fig. 1c. The apparent relative impairment (i.e., greater variability of correlation coefficients) visible in the septum in the bright-blood data Fig. 1c—bottom is erroneously caused by blood flow artifacts in phase-encoding direction (arrows in panel 1b′).

### Reproducibility

Linear regression analysis was used to assess the agreement between velocities obtained by black-blood versus bright-blood methodologies and to assess reproducibility of the black-blood data following repeated acquisitions. Comparisons were made by calculating mean velocities averaged over the entire LV segmentation mask for all time frames, and the slopes and correlation coefficients are listed in Table 1.

**Table 1:** Slopes and Correlation Coefficients from the Regression Analysis

	Bright- versus black-blood		Black- versus black-blood	
		
	Slope	*r*	Slope	*r*
*v*_r_	0.80*a	0.97	1.06#b	0.99
*v*_φ_	0.77*a	0.81	0.97	0.98
*v*_z_	0.84*a	0.87	0.99	0.97

* a*P* < 0.01.

#b *P* < 0.02.

**Fig. 2:**
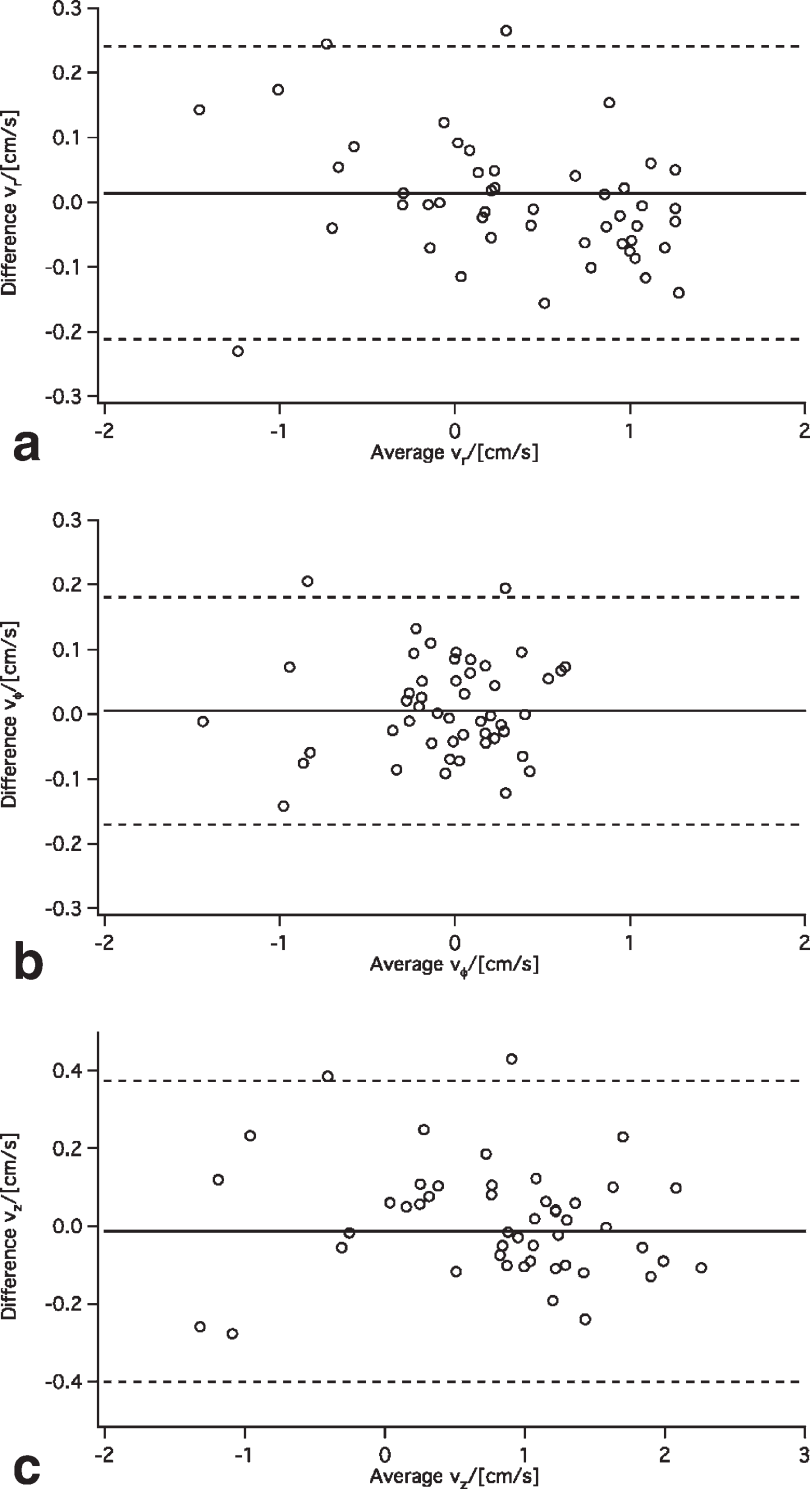
Bland–Altman plots for (**a**) radial, (**b**) tangential, and (**c**) longitudinal velocities, obtained in repeated measurements in a mid-ventricular slice with black-blood contrast (*n* = 3). The solid line represents the bias, the dashed lines ±2 SD confidence interval.

Excellent reproducibility for these data was also confirmed by the Bland–Altman-analysis shown in Fig. 2. It can be observed in Table 2 that the repeated black-blood scans provided two to six times smaller biases with a decreased scatter than the bright- versus black-blood scans, resulting in tighter limits of agreements.

**Table 2:** Biases from Bland–Altman Analysis

	Bright- versus black-blood (agreement)	Black- versus black-blood (reproducibility)
Number of animals	6	3
Total number of data points	119	50
*v*_r_ in (cm/s)	0.05 ± 0.27	0.01 ± 0.11
*v*_φ_ (in cm/s)	−0.01 ± 0.19	0.005 ± 0.088
*v*_z_ in (cm/s)	0.06 ± 0.32	−0.01 ± 0.19

### Application to Control Hearts

**Fig. 3:**
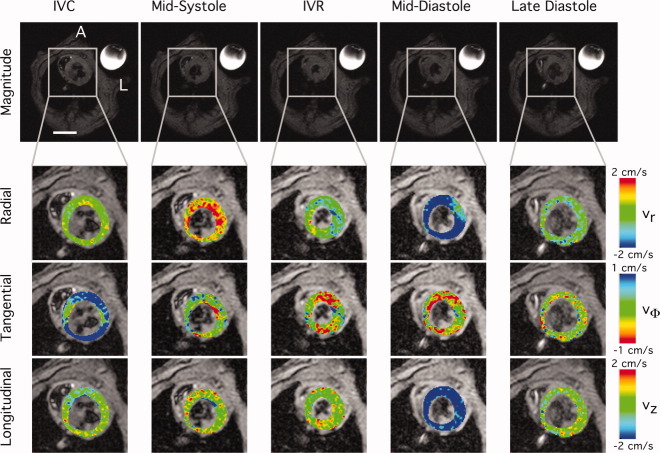
Mid-ventricular magnitude images (upper row), and color-coded maps of radial (second row), tangential (third row), and longitudinal velocities (bottom row), obtained at five different time points throughout the cardiac cycle (IVC/IVR—isovolumetric contraction/relaxation). Please note that the color-map for the tangential velocity is inverted relative to the radial/longitudinal velocities to match clinical convention as in Ref.^26^. The anatomical images in the top row also shows the agarose reference in the top right corner of each panel. A, anterior; L, left. Scale bar: 5 mm.

Of the 22 mice scanned, one was excluded due to gradient interference with the ECG and subsequent mistriggering. Figure 3 shows anatomical images (top row) across the entire mouse acquired on a mid-ventricular level. Five characteristic cardiac frames are shown: early systole (during isovolumetric contraction; Fig. 3a), mid-systole (Fig. 3b), early diastole (during isovolumetric relaxation; Fig. 3c), mid-diastole (Fig. 3d), and late diastole (Fig. 3e). Corresponding color-coded maps are shown for radial (second row), tangential (third row), and longitudinal velocities (bottom row), respectively. During early systole, in-plane motion is mainly determined by a clockwise rotation (positive tangential velocities), which precedes LV contraction in mid-systole as indicated by high-radial velocity components. Longitudinal motion is less pronounced and more evenly distributed across the systolic phase but has a clear minimum in mid-diastole, which coincides with relaxation of the left ventricle (negative radial velocities). This again is preceded by a counterclockwise rotation starting in early diastole, and lasting until mid-diastole. No significant motion was detected in late diastole in any of the components (Fig. 3e).

**Fig. 4:**
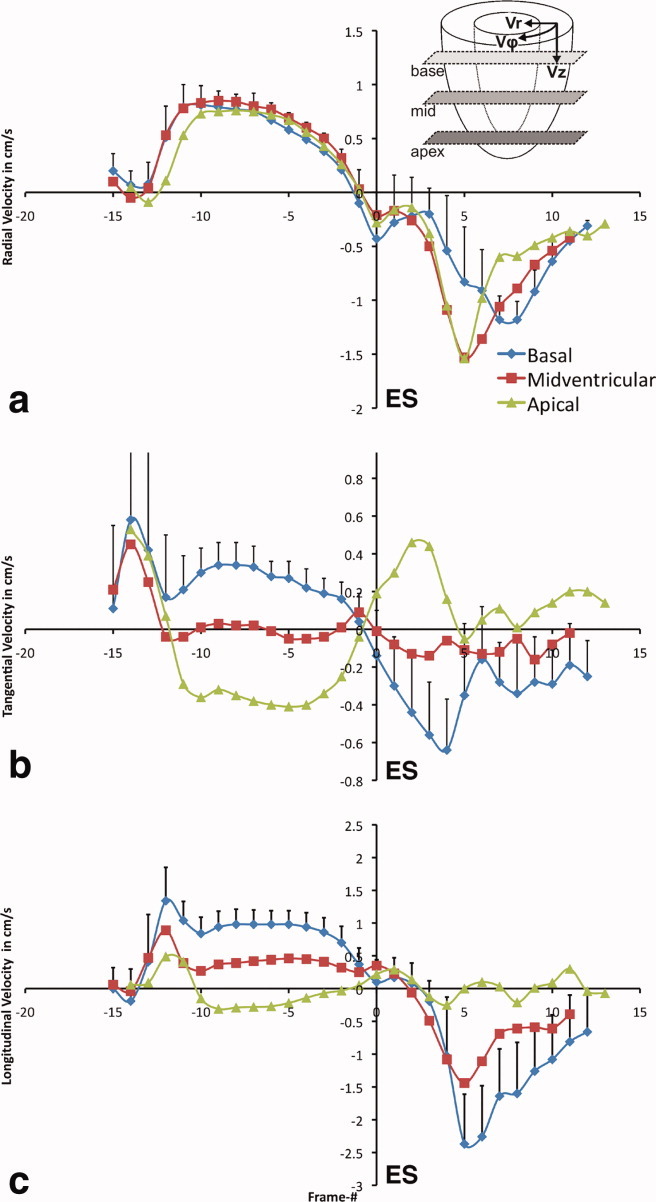
Time courses of mean (**a**) radial, (**b**) tangential, and (**c**) longitudinal velocities with high-temporal resolution averaged over all mice. Each graph was normalized to end-systole and shows a comparison of data for basal, midventricular, and apical slice locations as indicated in the insert in (a). The error bars indicate the standard deviation over all mice and are shown for the basal velocities only. [Color figure can be viewed in the online issue, which is available at wileyonlinelibrary.com.]

In Figure 4, the global velocity time courses averaged over all mice are shown. The error bars, which indicate the standard deviation over all mice, are shown for the basal velocities only to maintain clarity of the plots. Particularly, the radial velocity depicts contraction/expansion (Fig. 4a), *v*_φ_ describes the rotational motion (Fig. 4b), and the longitudinal velocity indicates lengthening/shortening (Fig. 4c) of the left ventricle. Each graph shows the motion patterns for basal, mid-ventricular, and apical slices, normalized to the respective end-systolic frame. The insert in Fig. 4a illustrates the location of the three slices relative to the entire ventricle. Global radial velocities are similar, while tangential and longitudinal motion patterns are distinctively different between the three slices. Not surprisingly, most of the rotation occurs in the basal and the apical slices, which rotate opposite relative to each other. Notably, the apical slice starts to rotate clockwise at early systole (as do basal and mid-ventricular slices) and changes direction in mid-systole. Most of the longitudinal motion can be found in the basal slice, which moves toward the apex in systolic phase. Furthermore, all three slices showed the same pattern at early systole, i.e., an initial displacement toward the apex.

**Fig. 5:**
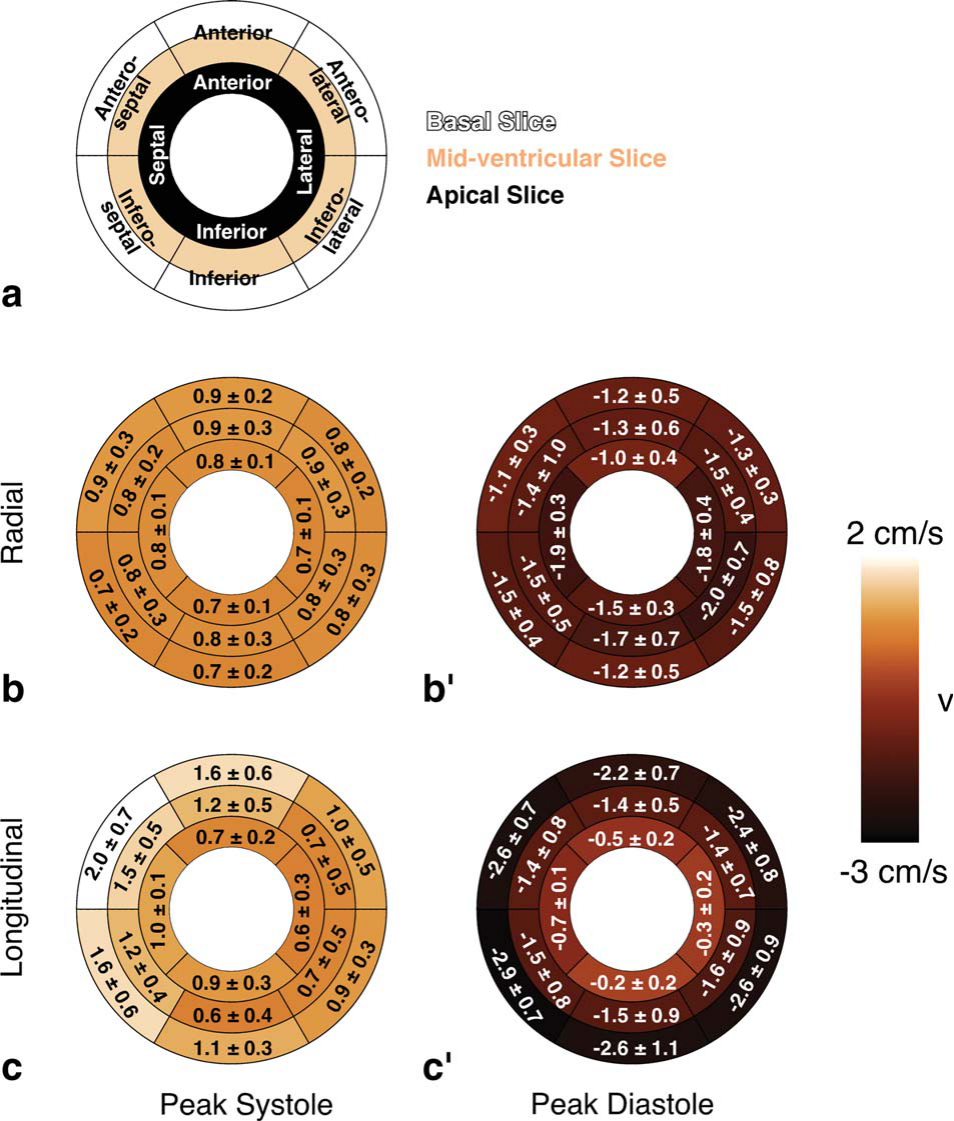
**a**: Schematics illustrating the partitioning of the left ventricle six regions of interest for the basal/midventricular slice and four regions of interest for the apical slice. Bull's eye plots for (**b**) radial and (**c**) longitudinal velocities obtained at peak systole (as defined by the maximum velocities during systole) according to the American Heart Association 16-segment model. b′,c′: Corresponding velocities at peak diastole (as identified by the minimum velocities during diastole). The colors encode the average over all animals, and the numbers correspond to mean ± SD. [Color figure can be viewed in the online issue, which is available at wileyonlinelibrary.com.]

Bull's eye plots are depicted in Fig. 5, graphically illustrating regional peak velocities for radial (Fig. 5b,b′) and longitudinal (Fig. 5c,c′) velocities in 16-segments according to the American Heart Association convention (mean ± SD). Figure 5b,c corresponds to peak-systole, while Fig. 5b′,c′ indicates peak diastole, all of which are defined by maximum (systole)/minimum (diastole) velocities. Figure 5a illustrates the segment location in the LV for basal/mid-ventricular and apical slices, respectively. It can be seen that radial peak velocities are similar throughout the entire left-ventricle with 0.82 ± 0.07 cm/s in systole and slightly more heterogeneous in diastole with −1.5 ± 0.3 cm/s. Figure 5c,c′ confirms that mostly basal and mid-ventricular slices exhibit longitudinal motion. While longitudinal peak velocities in diastole are quite homogeneously distributed across each slice, both septal regions and the anterior wall show higher systolic peak velocities compared with the lateral and inferior walls.

**Fig. 6:**
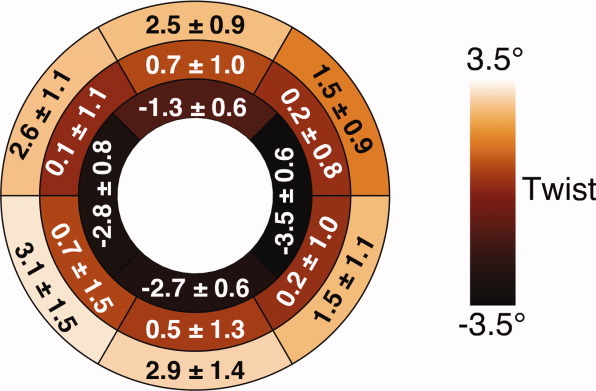
Bull's eye plot illustrating the mean regional twist, obtained from the tangential velocities. The region of interest definition follows Fig. 5a. The colors encode the average over all animals, and the numbers correspond to mean ± SD. [Color figure can be viewed in the online issue, which is available at wileyonlinelibrary.com.]

The bull's eye plot for rotational motion, characterized by the regional twist, is shown in Fig. 6. The apex turns counterclockwise (negative twist), whereas the basal myocardium turns clockwise (positive twist). The mid-ventricular slice exhibits only a small twist throughout all segments of 0.42 ± 0.26° (min: 0.13°, max: 0.74°). This compares with 2.5 ± 0.5° in the base and −2.6° ± 0.9° in the apical slice, respectively. Based on the twist, the ventricular torsion was calculated for four segments defined for the apical slice and is listed in Table 3. Largest torsion was found in the lateral, inferior, and septal walls, while the torsion in the anterior wall is only ∼60% compared with the other segments. Importantly, TPM also allowed for quantifying torsion in the epicardial and endocardial compartments of each segment, demonstrating that the epicardium consistently exhibits less torsion than the endocardium. While the septal compartment showed the largest torsion within the epicardial segments (−4.93° ± 0.45°), it was the lateral wall in the endocardium (−7.2° ± 1.3°).

**Table 3:** Torsion (Mean ± SD) (Defined as Apical Twist – Basal Twist)

	Septum	Inferior	Lateral	Anterior
Overall /[(°)]	−5.54 ± 0.81	−5.7 ± 0.64	−5.68 ± 0.77	−3.42 ± 0.86
Epi/[ (°)]	−4.93 ± 0.45	−4.72 ± 0.64	−4.60 ± 0.48	−2.60 ± 0.76
Endo /[(°)]	−6.5 ± 1.4	−7.0 ± 1.1	−7.2 ± 1.3	−4.6 ± 1.3

Global cardiac functional parameters were within published values for normal mice. Particularly, we measured for: LV-mass: 74.1 ± 4.6 mg, end-diastolic volume: 45.8 ± 6.9 μL, end-systolic volume: 12.6 ± 4.9 μL, SV: 33.2 ± 5.2 μL, ejection fraction: 72.9 ± 8.2%, heart rate 468 ± 33 bpm, and cardiac output: 15.5 ± 2.4 mL/min (mean ± SD, *n* = 21).

### Application to IR Model

**Fig. 7:**
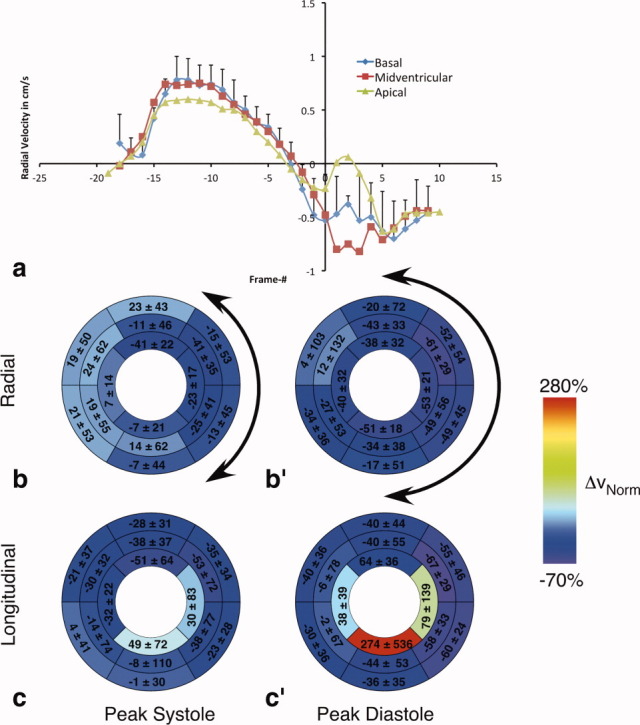
Results for the ischemia–reperfusion mouse model. **a**: Time course of mean radial velocities averaged over all IR mice. Analogously to Fig. 4a, each graph was normalized to end-systole and shows a comparison of data for basal, midventricular, and apical slice locations. The error bars indicate the standard deviation over all mice and are shown for the basal velocities only. Bull's eye plots for normalized (**b**) radial and (**c**) longitudinal velocity differences obtained at peak systole (as defined by the maximum velocities during systole) according to the American Heart Association 16-segment model and calculated according to Eq. 1. (b′,c′) Corresponding velocity differences at peak diastole (as identified by the minimum velocities during diastole). The colors encode the average difference (%) between mean peak velocities of IR (*n* = 5) and control hearts (*n* = 21) normalized to the control hearts. The numbers correspond to mean ± SD. The arrows in panels (b) and (b′) indicate the injured area, mainly corresponding to the anterolateral/inferolateral sections. [Color figure can be viewed in the online issue, which is available at wileyonlinelibrary.com.]

In Figure 7a, the global velocity time courses averaged over the five mice subjected to 45 min of ischemia followed by 24 h of reperfusion are shown. The error bars, which indicate the standard deviation over all mice, are again only shown for the basal velocities. Regional analysis of the peak systolic and diastolic velocity components, which were normalized to the respective velocities in the corresponding regions of interest of the control hearts, revealed reduced contraction/longitudinal motion in the injured areas [indicated by arrows in panels (b,b′)], which are partially compensated by higher velocities in the remote zone.

## DISCUSSION

The aim of this article was to establish and validate phase-contrast MRI as a tool that can be used routinely on conventional preclinical MR systems to provide a comprehensive assessment of regional cardiac function in mouse hearts. TPM is only one out of three MR methods that can be used to characterize transmural wall motion in mice. The advantages and disadvantages of TPM compared with tagging and (single time-frame) DENSE have been discussed in Ref.^17^. More recently, DENSE has been extended to allow for multiframe application covering the entire RR interval, albeit at somewhat lower temporal resolution than we achieved for TPM. Our TPM method was optimized to provide an acceptable balance between temporal/spatial resolution and scan time, as TPM is rarely used as stand-alone method but in combination with other MR phenotyping techniques (such as cine-MRI for global cardiac function assessment, contrast-enhanced MR for infarct size measurement, etc.). The scan time of less than 15 min for the three slices makes this method suitable for routine application.

The implemented method differed from the previously published work (17) in timing, maximum encoded velocity (venc), encoding scheme, spatial resolution, and postprocessing. Echo and repetition times were shorter in our case, despite smaller venc's and the echo position in the middle of the acquisition window. High-temporal resolution can be considered to be more important for the TPM technique than very high-spatial resolution due to averaging within segments. The more than 2-fold improved in-plane resolution and ∼30% thinner slices achieved in Ref.^17^ were most likely possible due to the ∼2-fold higher field strength and the volume coil with a ∼40% smaller inner diameter. However, in our experience, it is impossible (at least on horizontal-bore magnets) to fit adult mice in such a small volume without compromising animal physiology such as compression of thorax and restriction of normal breathing patterns. Nevertheless, sufficient signal-to-noise ratio and spatial resolution was obtained with our method to accurately detect differences in epicardial and endocardial motion patterns as demonstrated in Table 3. Furthermore, optimized venc's provide better resolution of velocities and reduced phase noise (29). TPM sequences are very gradient-demanding and can generate significant heat, which may transfer to the animal, and, therefore, affect animal physiology (depending on gradient duty cycle performance and efficiency of animal heating system). Hence, any changes in physiology during the experiment, which potentially may affect transmural wall motion, will average out with the interleaved encoding scheme. Finally, only four scans were acquired instead of seven as in Ref.^17^. Velocity information was obtained by phase subtraction rather than by linear fitting of ±v-encoded and motion compensated scans, nearly halving the required scan time. Our approach compensates for erroneous phase contributions caused by *B*_0_ inhomogeneities, off-resonance effects, etc. Linear background phase errors were corrected further using information from an external, static agarose reference, which provided high signal-to-noise ratio due to short *T*_1_ times (22).

A second aspect of the optimization was to determine whether or not black-blood contrast is beneficial for transmural wall motion assessment in the mouse, as shown for human hearts (19, 20). Black-blood contrast necessitates the application of dedicated blood suppression techniques (i.e., double inversion or saturation), typically applied at the end or the beginning of the cine-train, which is omitted for bright-blood contrast. While bright-blood techniques provide extended coverage of the cardiac cycle, the total duration of blood-saturation module with 7.5 ms and a “safety delay” of typically 10–15 ms (to compensate for changes of heart rate during the scan), was still short enough to cover all relevant parts of the cardiac cycle. This “dead-time” of ∼20 ms can potentially represent a problem for extremely high heart rates, as typically observed in pharmacologically induced stress studies. In this case, the black-blood module could be applied at the beginning of the cine-train and followed by data acquisition over at least 1.1 times the cardiac cycle length as suggested by Ref.^16^. Two of six mice showed considerable flow-related artifacts in the motion-encoding bright-blood acquisitions, which were not present in the black–black scans. Exclusion of these two mice from the regression analysis still did not give the same reproducibility in myocardial velocities as the repeated black-blood scans (data not shown). Importantly, bright-blood contrast was found to impact on absolute velocities (the slopes in the regression analysis deviated significantly from one—Table 1), increased biases and larger confidence intervals in the Bland–Altman analysis (Table 2), and motion pattern as demonstrated by the correlation coefficient plots in Fig. 1. Differences between black-blood and bright-blood contrast scans are only meaningful, if the reference technique (i.e., the black-blood acquisition) is reproducible as demonstrated in Tables 1 and 2 and Fig. 2. The benefit of black-blood contrast is of relevance particularly for diseased hearts, which may have less stable heart rates during data acquisition, and—in our experience—may therefore be more prone to these artifacts.

The main aim of this article was to provide detailed global and regional left-ventricular motion patterns. While twist and torsion angles have been reported before for mouse hearts, this study provides for the first time a detailed motion analysis of the entire cardiac cycle in normal, adult C57Bl/6 mice under isoflurane anesthesia using (phase-contrast) MRI, and followed clinical convention for both the different velocity components and the segmental analysis (21, 26). While Herold et al. only provided twist angles to characterize in-plane motion, our study demonstrates a complex motion pattern between basal, mid-ventricular, and apical slices as well as within the time course for each slice as shown for the radial and tangential velocity components (Fig. 4a,b). Maximum longitudinal velocities were measured by Herold et al. (referred to as axial velocity in Ref.^17^) in the systolic and the diastolic phase for four segments at equivalent slice positions. While their study used the opposite sign convention to our study, it has to be noted that the longitudinal motion pattern was different. Importantly, they observed a symmetric movement around the mid-ventricular slice, where basal and apical segments exhibited similar velocity magnitudes but opposite directions. In our study, longitudinal movement was mainly from base to apex, where the apex only showed minor motion toward the base (as characterized by small velocities in *z*-direction). It cannot be excluded that this difference is caused by the vertical orientation of the mouse in the magnet used in Ref.^17^. We have previously shown that positioning mice upright in a vertical-bore magnet causes a mild orthostatic effect (30), but the potential impact on transmural wall motion warrants further investigation. Unfortunately, the studies by Streif et al. (16) and Nahrendorf et al. (18) applied TPM in a single (mid-ventricular) slice only and did not assess longitudinal velocities. Moreover, longitudinal motion patterns have been shown to be abnormal in patients with LV hypertrophy (26).

Twist angles and torsion were similar to the values reported by Henson et al. (31) and, more recently, by Zhong and Yu (15), using tagging and DENSE. However, apical twist was about 4-fold smaller than the one derived from phase-contrast MRI in Ref.^17^, which also observed the reduced torsion in the anterior wall. Differences in epicardial and endocardial torsions have been shown in humans (32,33), pigs (34), and rats (35) using echocardiography, MR tagging, or DENSE. Moreover, strain variation from epicardium to endocardium obtained from tagged mouse heart data has been shown previously (11, 36). However, to the best of our knowledge, this is the first (MR) study that derived epicardial and endocardial motion patterns in mouse hearts from phase-contrast MRI. Specifically, we found a gradient in the transmural twist magnitudes between epicardium and endocardium as reported for humans (32, 33) and dogs (37). Twist and torsion of the left ventricle is generated by the different regional orientations of myofibres, which undergo a smooth transition from a right-handed helical structure in the subendocardial tissue to a left-handed helical arrangement in epicardium in normal myocardium (e.g., see Ref.^38^ for mouse hearts). Therefore, the ability to perform a regional analysis of left-ventricular rotational behavior in these compartments adds another valuable tool for a comprehensive phenotyping characterization of murine hearts. The spatial resolution of 200 μm of our TPM data is still superior to the improved tagging resolution of 300 μm reported by Zhong et al. (36).

The proof-of-principle application to the ischemia–reperfusion mouse model (Fig. 7) demonstrated that the mean global (magnitude) velocities were generally smaller than for control mice (Fig. 4a). Not only were the apical velocities notably smaller than the basal/mid-ventricular components in the systolic part of the cardiac cycle, the motion pattern in the diastolic part differed also considerably from the one of control hearts shown in Fig. 4a. The large standard deviations calculated for the longitudinal velocities in the apical regions of interest are mainly a result of the normalization process. Black-blood contrast was not found to be inferior compared with normal hearts.

In conclusion, this work presents an optimized method based on phase-contrast MRI that allows for a comprehensive assessment of regional cardiac function in mouse hearts at 9.4 T. Black-blood contrast was found to be essential to obtain accurate and reproducible velocity encoding. A detailed analysis was performed to establish baseline values of normal transmural wall motion pattern. This technique will help to investigate systolic and diastolic functions in genetically and surgically manipulated mouse models of human heart disease.
